# Antioxidant activities of traditional plants in Sri Lanka by DPPH free radical-scavenging assay

**DOI:** 10.1016/j.dib.2018.02.013

**Published:** 2018-02-10

**Authors:** Kotaro Hara, Takao Someya, Katsura Sano, Yoshimasa Sagane, Toshihiro Watanabe, R.G.S. Wijesekara

**Affiliations:** a ALBION Co. Ltd., 1-7-10 Ginza, Chuo-ku, Tokyo 104-0061, Japan; b Department of food and Cosmetic Science, Faculty of Bioindustry, Tokyo University of Agriculture, 196 Yasaka, Abashiri, Hokkaido 099-2493, Japan; c Department of Aquaculture and Fisheries, Faculty of Livestock, Fisheries and Nutrition, Wayamba University of Sri Lanka, Makandura, Gonawila 60170, Sri Lanka

**Keywords:** Antioxidative activity, DPPH radical-scavenging assay, Traditional plant, Medical herb

## Abstract

This article describes free radical-scavenging activities of extracts of several plants harvested in Sri Lanka through the 1,1-diphenyl-2-picrylhydrazyl (DPPH) assay. These plants have traditionally been used in the indigenous systems of medicine in Sri Lanka, such as Ayurveda, as described below. (English name, “local name in Sri Lanka,” (scientific name)).

bougainvillea plant, “bouganvilla,” (*Bougainvillea grabla*), purple fruited pea eggplant,”welthibbatu,” (*Solanum trilobatum*) [1], country borage plant, “kapparawalliya,” (*Plectranthus amboinicus*) [2], malabar nut plant, “adhatoda,” (*Justicia adhatoda*) [3], long pepper plant,”thippili,” (*Piper longum*) [4], holy basil plant, “maduruthala,” (*Ocimum tenuiflorum*) [5], air plant, “akkapana,” (*Kalanchoe pinnata*) [6], plumed cockscomb plant, “kiri-henda,” (*Celosia argentea*) [7], neem plant,”kohomba,” (*Azadirachta indica*) [8], balipoovu plant, “polpala,” (*Aerva lanata*) [9], balloon-vine plant, “wel penera,” (*Cardiospermum halicacabum*) [10], emblic myrobalan plant, “nelli,” (*Phyllanthus emblica*) [11], indian copperleaf plant, “kuppameniya,” (*Acalypha indica*) [12], spreading hogweed plant, “pita sudu sarana,” (*Boerhavia diffusa*) [13], curry leaf plant, “karapincha,” (*Murraya koenigii*) [14], indian pennywort plant, “gotukola,” (*Centera asiatica*) [15], jewish plum plant, “ambarella,”(*Spondias dulcis*) [16].

**Specifications Table**

**Table:** 

Subject area	*Biology*
More specific subject area	*Cell biology*
Type of data	*Graph*
How data was acquired	*Fluorescent microscope (SpectraMax® i3x, MOLECULAR DEVICES)*
Data format	*Analyzed*
Experimental factors	*1,1-diphenyl-2-picrylhydrazyl*
Experimental features	*Analysis of free radical-scavenging activity through the DPPH assay*
Data source location	*Negombo, Sri Lanka*
Data accessibility	*Data are available within this article*

**Value of the data**

• Data represent free radical-scavenging activities of extracts of several plants, and support further studies for estimating their biological effects.
• These data indicate that several plants exhibit antioxidant activities and could be further investigated for their use as pharmacologic and cosmetic agents.
• This article investigates biological effects of plants traditionally used in indigenous systems of medicine.

## 1 Data

This data article contains bar graphs showing anti-oxidative activities of several plants extracts, harvested in Negombo, Sri Lanka. Anti-oxidative activities of each plants extracts were determined free radical scavenging activity of NLE through 1,1-diphenyl-2-picrylhydrazyl (DPPH) assay. The data represent the mean ± SE values from triplicate independent experiments (*P < 0.05, **P < 0.001 and ***P < 0.001 vs. control (0.0%)).

## 2 Experimental design, materials and methods

All plants were harvested from a medicinal garden at the Institute of Traditional Plants (Negombo, Sri Lanka). Each plant extract was prepared using specific solvents as described below.

bougainvillea plant, “bouganvilla,” (*Bougainvillea grabla*), flower, 70% EtOH; purple fruited pea eggplant,”welthibbatu,” (*Solanum trilobatum*), shoot, 70% EtOH; country borage plant, “kapparawalliya,” (*Plectranthus amboinicus*), leaf ,70% EtOH; malabar nut plant, “adhatoda,” (*Justicia adhatoda*), leaf, 70% EtOH; long pepper plant,”thippili,” (*Piper longum*), leaf, 70% EtOH; holy basil plant, “maduruthala,” (*Ocimum tenuiflorum*), shoot, 70% EtOH; air plant, “akkapana,” (*Kalanchoe pinnata*), leaf, 70% EtOH; plumed cockscomb plant, “kiri-henda,” (*Celosia argentea*), shoot, 70% EtOH; neem plant,”kohomba,” (*Azadirachta indica*), leaf, 50% BG; balipoovu plant, “polpala,”(*Aerva lanata*), shoot, 50% EtOH; balloon-vine plant, “wel penera,” (*Cardiospermum halicacabum*), shoot, 50% EtOH; emblic myrobalan plant, “nelli,” (*Phyllanthus emblica*), leaf 70% EtOH; indian copperleaf plant, “kuppameniya,” (*Acalypha indica*), shoot, 50% EtOH; spreading hogweed plant, “pita sudu sarana,” (*Boerhavia diffusa*), shoot, 70% EtOH; curry leaf plant, “karapincha,” (*Murraya koenigii*), leaf, 70% EtOH; indian pennywort plant, “gotukola,” (*Centera asiatica*), shoot, 70% EtOH; jewish plum plant, “ambarella,”(*Spondias dulcis*), fruit, 70% EtOH; jewish plum plant, “ambarella,”(*Spondias dulcis*), leaf, 70% EtOH.

## 3 1,1-diphenyl-2-picrylhydrazyl (DPPH) assay (assay for free radical-scavenging)

**Fig. 1:**
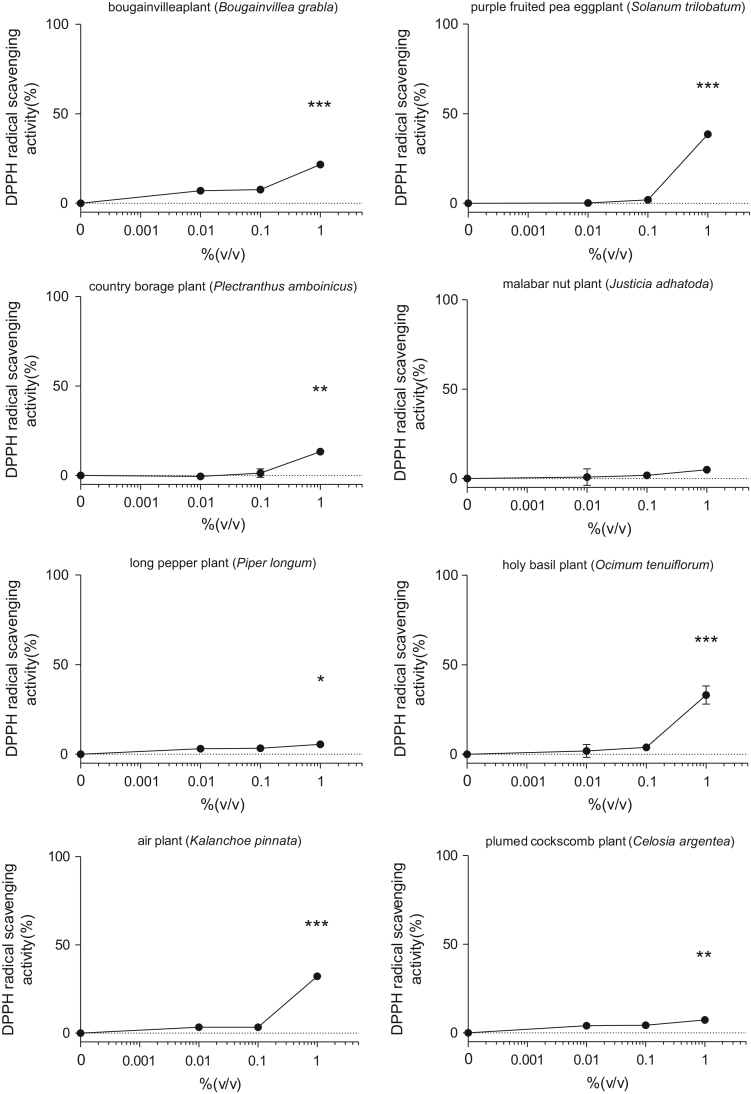

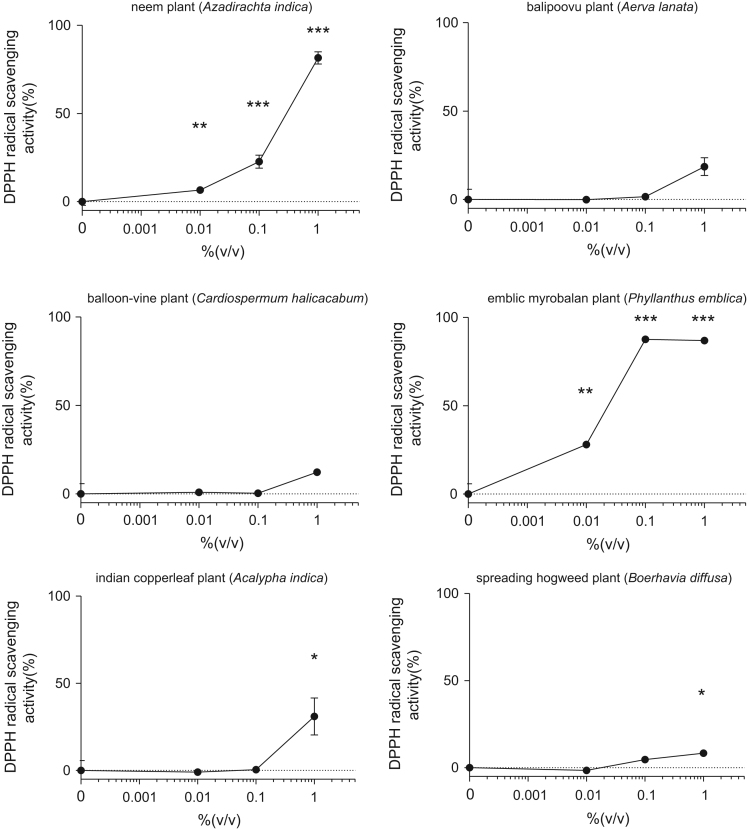

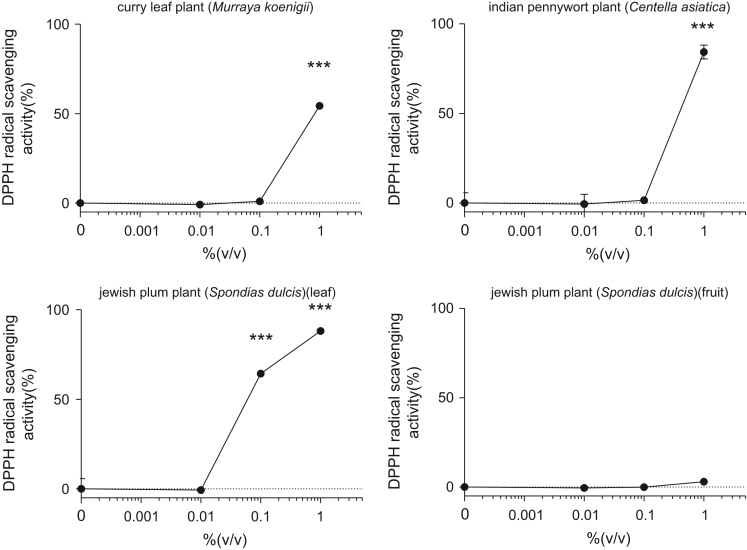
Free radical-scavenging activity of several plants extracts harvested in Sri Lanka through 1,1-diphenyl-2-picrylhydrazyl (DPPH) assay. The results are presented as a relative of the control (0%). The values are shown as the mean ± SE of three independent experiments.

The free radical scavenging capacity of plants extracts were analyzed by using DPPH. Plants extracts were diluted with 0.1 M acetic acid buffer (pH 5.5) at various concentrations (0–1.0% (volume/volume)). Ascorbic acid (1–10 µg/ml) was used as a positive control. A volume of 40 μl of samples and 60 μl of ethanol (with or without 0.1 mM DPPH) were mixed in 96-well plate at room temperature for 30 minutes, and the absorbance at 517 nm (A517) was measured. The DPPH scavenging effect was calculated as follows: Scavenging effect (%) = 100-(A-Ab)/(A0-A0b) × 100, where A0:A517 of DPPH without sample, A0b: A517 without sample and DPPH, A: A517 of sample and DPPH, and Ab: A517 of sample without DPPH (Fig. 1).
