# Supplementary material for: Antioxidant activities of traditional plants in Sri Lanka by DPPH free radical-scavenging assay
Source: Data Brief. 2018 Feb 10;17:870–5. doi: 10.1016/j.dib.2018.02.013 (PMC5834651; doi:10.1016/j.dib.2018.02.013)
Supplement: Supplementary file 1 — Supplementary material [file mmc1.docx]

**Conflicts of interests:** None
